# CasTabDetectoRS: Cascade Network for Table Detection in Document Images with Recursive Feature Pyramid and Switchable Atrous Convolution

**DOI:** 10.3390/jimaging7100214

**Published:** 2021-10-16

**Authors:** Khurram Azeem Hashmi, Alain Pagani, Marcus Liwicki, Didier Stricker, Muhammad Zeshan Afzal

**Affiliations:** 1 Department of Computer Science, Technical University of Kaiserslautern, 67663 Kaiserslautern, Germany; didier.stricker@dfki.de; 2 Mindgarage, Technical University of Kaiserslautern, 67663 Kaiserslautern, Germany; 3 German Research Institute for Artificial Intelligence (DFKI), 67663 Kaiserslautern, Germany; alain.pagani@dfki.de; 4 Department of Computer Science, Luleå University of Technology, 971 87 Luleå, Sweden; marcus.liwicki@ltu.se

**Keywords:** table detection, table recognition, cascade Mask R-CNN, atrous convolution, recursive feature pyramid networks, document image analysis, deep neural networks, computer vision, object detection

## Abstract

Table detection is a preliminary step in extracting reliable information from tables in scanned document images. We present CasTabDetectoRS, a novel end-to-end trainable table detection framework that operates on Cascade Mask R-CNN, including Recursive Feature Pyramid network and Switchable Atrous Convolution in the existing backbone architecture. By utilizing a comparativelyightweight backbone of ResNet-50, this paper demonstrates that superior results are attainable without relying on pre- and post-processing methods, heavier backbone networks (ResNet-101, ResNeXt-152), and memory-intensive deformable convolutions. We evaluate the proposed approach on five different publicly available table detection datasets. Our CasTabDetectoRS outperforms the previous state-of-the-art results on four datasets (ICDAR-19, TableBank, UNLV, and Marmot) and accomplishes comparable results on ICDAR-17 POD. Upon comparing with previous state-of-the-art results, we obtain a significant relative error reduction of 56.36%, 20%, 4.5%, and 3.5% on the datasets of ICDAR-19, TableBank, UNLV, and Marmot, respectively. Furthermore, this paper sets a new benchmark by performing exhaustive cross-datasets evaluations to exhibit the generalization capabilities of the proposed method.

## 1. Introduction

**Figure 1:**
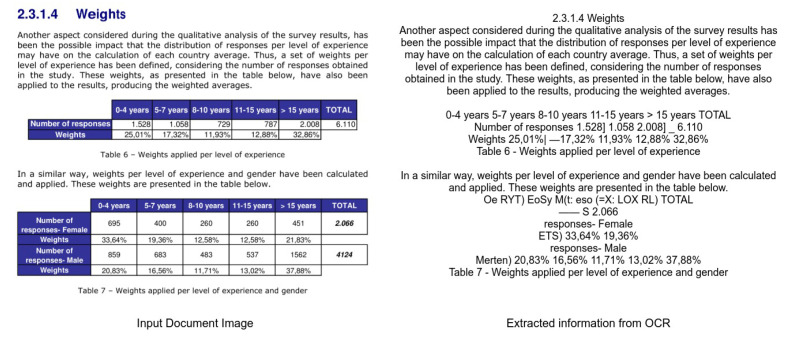
Illustrating the need of applying table detection before extracting information in document images. We apply open source Tesseract-OCR [10] on a document image containing two tables. Besides the textual content, the OCR system fails miserably in interpreting information from tables.

The process of digitizing documents has received significant attention in various domains, such as industrial, academic, and commercial sectors. The digitization of documents facilitates the process of extracting information without manual intervention. Apart from the text, documents contain graphical page objects, such as tables, figures, and formulas [1,2]. Albeit modern Optical Character Recognition (OCR) systems [3,4,5] can extract the information from scanned documents, they fail to interpret information from graphical page objects [6,7,8,9]. Figure 1 exhibits the problem of extracting tabular information from a document by applying open-source Tesseract OCR [10]. It is evident that even the state-of-the-art OCR system fails to parse information from tables in document images. Therefore, for complete table analysis, it is essential to develop accurate table detection systems for document images.

The problem of accurate table detection in document images is still an open problem in the research community [8,11,12,13,14]. The high amount of intra-class variance (arbitraryayouts of tables, varying presence of rulingines) andow amount of inter-class variance (figures, charts, and algorithms equipped with horizontal and verticalines thatookike tables) makes the task of classifying andocalizing tables in document images even more challenging. Owing to these involved intricacies in table detection, custom heuristics based methodsack in producing robust solutions [15,16].

Prior works have tackled the involved challenges of table detection througheveraging meta-data or utilizing morphological information from tables. However, these methods are vulnerable in case of scanned document images [17,18]. Later, the utilization of deepearning-based approaches to attempt the task of table detection in document images have shown a remarkable improvement in the past few years [8]. Intuitively, the task of table detection has been formulated as an object detection problem [7,19,20,21], in which a table can be a targeted object present in a document image instead of a natural scene image. Consequently, the rapid progress in object detection algorithms hased to the extraordinary improvement in state-of-the-art table detection systems [11,12,13,20]. However, the prior approaches struggle in predicting preciseocalization of tabular boundaries in distinctive datasets. Moreover, they either rely on external pre-/post-processing methods to further refine their predictions [11,13] or incorporate memory intensive deformable convolutions [12,20]. Furthermore, prior state-of-the-art methods relied on heavy and high resolution backbones, such as ResNeXt-101 [22] and HRNet [23], which require expensive process of training.

To tackle the aforementioned issues present in existing approaches, we present CasTabDetectoRS, an end-to-end trainable novel object detection pipeline by incorporating the idea of Recursive Feature Pyramids (RFP) and Switchable Atrous Convolutions (SAC) [24] into Cascade Mask R-CNN [25] for detection of tables in document images. Furthermore, this paper empirically establishes that generic and robust table detection systems can be built without depending on pre-/post-processing methods and heavy backbone networks.

To summarize, the main contribution of this work are explained below:

- We present CasTabDetectoRS, a novel deepearning-based table detection approach that operates on Cascade Mask R-CNN equipped with recursive feature pyramid and switchable atrous convolution.
- We experimentally deny the dependency of custom heuristics or heavier backbone networks to achieve superior results on table detection in scanned document images.
- We accomplish state-of-the-art results on four publicly available table detection datasets: ICDAR-19, TableBank, Marmot, and UNLV.
- We demonstrate the generalization capabilities of the proposed CasTabDetectoRS by performing the exhaustive cross-datasets evaluation.

The remaining paper is structured as follows. Section 2 categorizes the prioriterature into rule-based, earning-based, and object detection-based methods. Section 3 describes the proposed table detection pipeline by addressing all the essential modules, such as RFP (Section 3.1), SAC (Section 3.2), and Cascade Mask R-CNN (Section 3.3). Section 4 presents the comprehensive overview of employed datasets, experimental details, and evaluation criteria, along with quantitative and qualitative analysis that follows with a comparison with previous state-of-the-art results and cross datasets evaluation. Section 5 concludes the paper and outlines possible future directions.

## 2. Related Work

The problem of table detection in documents has been investigated over the past few decades [16,26]. Earlier, researchers employed rule-based systems to solve table detection [16,26,27,28,29]. Afterwards, researchers exploited statisticalearning, mainly machineearning-based approaches, which were eventually replaced with deepearning-based methods [7,8,11,12,19,20,30,31,32,33,34].

### 2.1. Rule-Based Methods

To the best of our knowledge, Itonori et al. [26] addressed the problem of table detection in document images by employing a rule-based method. The proposed approacheveraged the arrangements of text-blocks and position of rulingines to detect tables in documents. Chandran and Kasturi [27] proposed another method that operates on rulingines to resolve table detection. Similarly, Pyreddy and Croft [35] published a heuristics-based table detection method that first identifies structural elements from a document and then filters the table.

Researchers have defined tabularayouts and grammars to detect tables in documents [29,36]. The correlation of white spaces and vertical connected component analysis is employed to predict tables [37]. Another method that transforms tables present in HTML documents into aogical structure is proposed by Pivk et al. [36]. Shigarov et al. [18] capitalized the meta-data from PDF files and treated each word as a block of text. The proposed method restructured the tabular boundaries byeveraging bounding boxes of each word.

We direct our readers to References [15,16,38,39,40] for a thorough understanding of these rule-based methods. Although the prior rule-based systems detect tables in document havingimited patterns, they rely on manual intervention toook for optimal rules. Furthermore, they are vulnerable in producing generic solutions.

### 2.2. Learning-Based Methods

Similar to the field of computer vision, the domain of table analysis have experienced a notable progress after incorporatingearning-based methods. Initially, researchers investigate machineearning-based methods to resolve table detection in document images. Unsupervisedearning was implemented by Kieninger and Dengel [41] to improve table detection in documents. Later, Cesarini et al. [42] employed supervisedearning-based system to find tables in documents. Their system reforms document into MXY tree representation. Later, the method predicts the tables by searching for blocks that are surrounded with rulingines. Kasar et al. [43] proposed a blend of SVM classifier and custom heuristics [43] to resolve table detection in documents. Researchers have also explored the capabilities of Hidden Markov Models (HMMs) toocalize tabular areas in documents [44,45]. Even though machineearning-based approaches have alleviated the research for table detection in documents, they require external meta-data to execute reliable predictions. Moreover, they fail to obtain generic solutions on document images.

Analogous to the field of computer vision, the power of deepearning has made a remarkable impact in the field of table analysis in document images [2,8]. To the best of our knowledge, Hao et al. [46] introduced the idea of implementing Convolutional Neural Network (CNN) to identify spatial features from document images. The authors merged these features with the extracted meta-data to predict tables in PDF documents.

Although researchers have employed Fully Convolutional Network (FCN) [47,48] and Graph Neural Network (GNN) [34,49] to perform table detection in document images, object detection-based approaches [7,8,11,12,19,20,30,31,32,33,34] have delivered state-of-the-art results.

### 2.3. Table Detection as an Object Detection Problem

There has been a direct relationship with the progress of object detection networks in computer vision and table detection in document images [8]. Gilani et al. [19] formulated the problem of table detection as an object detection problem by applying Faster R-CNN [50] to detect tables in document images. The presented work employed distance transform methods to modify pixels in raw document images fed to the Faster R-CNN.

Later, Schreiber et al. [7] presented another method that exploits Faster R-CNN [50] equipped with pre-trained base networks (ZFNet [51] and VGG-16 [52]) to detect tables in document images. Furthermore, Siddiqui et al. [20] published another Faster R-CNN-based method equipped with deformable convolutions [53] to address table detection having arbitraryayouts. Moreover, in Reference [33], the authors employed Faster R-CNN with a coronerocating an approach to improve the predicted tabular boundaries in document images.

Saha et al. [54] empirically established that Mask R-CNN [55] produces better results as compared to Faster R-CNN [50] in detecting tables, figures, and formulas. Zhong et al. [56] presented a similar conclusion by applying Mask R-CNN toocalize tables. Moreover, YOLO [57], SSD [58], and RetinaNet [59] have been employed to exhibit the benefits of closed domain fine-tuning on table detection in document images.

Recently, researchers have incorporated novel object detection algorithms, such as Cascade Mask R-CNN [25] and Hybrid Task Cascade (HTC) [60], to alleviate the performance of table detection systems in document images [11,12,13,14]. Although these prior methods have progressed state-of-the-art results, there is significant room for improvement inocalizing accurate tabular boundaries in scanned document images. Furthermore, the existing table detection methods either rely on heavier backbones or incorporate memory-intensive deformable convolutions. However, this paper proposes that state-of-the-art results can be achieved on table detection in scanned document images with intelligent incorporation of a relatively smaller backbone network with recursive feature pyramid networks and switchable atrous convolutions.

## 3. Method

**Figure 2:**
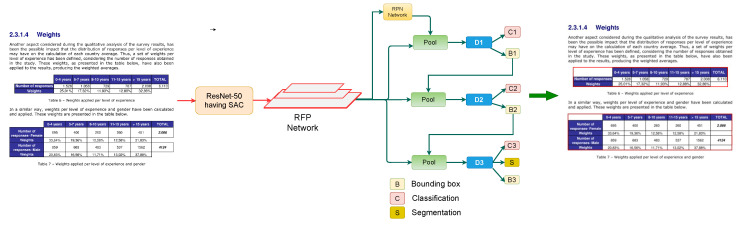
Presented table detection framework consisting of Cascade Mask R-CNN, incorporating RFP and SAC in backbone network (ResNet-50). The modules RFP and SAC are illustrated in separate figures.

The presented approach incorporates RFP and SAC into a Cascade Mask R-CNN to attempt table detection in scanned document images as exhibited in Figure 2. Section 3.1 discusses the RFP module, whereas Section 3.2 talks about SAC module. Section 3.3 describes the employed Cascade Mask R-CNN, along with complete description of the proposed pipeline.

### 3.1. Recursive Feature Pyramids

**Figure 3:**
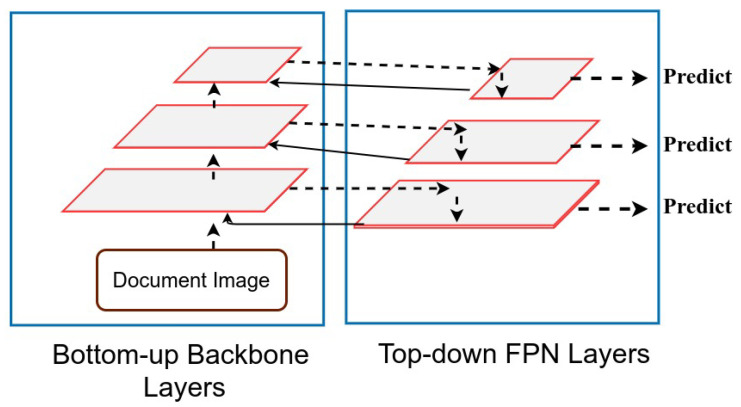
Illustrating design of Recursive Feature Pyramid module. The Recursive Feature Pyramid includes feedback connections that are highlighted with solidines. The top-down FPNayers send the feedback to the bottom-up backboneayers by inspecting the image twice.

Instead of the traditional Feature Pyramid Networks (FPN) [61], in our table detection framework, we incorporate Recursive Feature Pyramids (RFP) [24] to improve the processing of feature maps. To understand the conventional FPN,et $N_{j}$ denote the *j*-th stage of a bottom-up backbone network, and $F_{j}$ represent the *j*-th top-down FPN function. The backbone network *N* having FPN produces a set of feature maps, where total feature maps are equal to the number of stages. For instance, a backbone network with three stages is demonstrated in Figure 3. Therefore, with a number of stages *S* = 3, the output feature $f_{j}$ is given by:

(1) $$f_{j}=F_{j}\left(f_{j+1},i_{j}\right),\;\;i_{j}=N_{j}\left(i_{j-1}\right),$$

where *j* iterates over 1, …, S, $i_{0}$ represents the input image, and $f_{S+1}$ is set to 0. However, in the case of RFP, feedback connections are added to the conventional FPN, as illustrated in Figure 3 with solid black arrows. If we include feature transformations $T_{j}$ before joining the feedback connections from FPN to the bottom-up backbone, then, the output feature $f_{j}$ of RFP is explained in Reference [24] as:

(2) $$f_{j}=F_{j}\left(f_{j+1},i_{j}\right),\;\;i_{j}=N_{j}\left(i_{j-1},T_{j}\left(f_{j}\right)\right),$$

where *j* enumerates over S, and the transformation of FPN to RFP makes it a recursive function. If we unfold the RFP to a sequence of T, mathematically, it is given by:

(3) $$f_{j}^{t}=F_{j}^{t}\left(f_{j+1}^{t},i_{j}^{t}\right),\;\;i_{j}^{t}=N_{j}^{t}\left(i_{j-1}^{t},T_{j}^{t}\left(f_{j}^{t}\right)\right),$$

where *t* enumerates over *U*, and *U* is the number of unfolded steps. The superscript *t* represents the function and the features at unfolded step *t*. We empirically set *U* = 2 in our experiments. For a comprehensive explanation of the RFP module, please refer to Reference [24].

### 3.2. Switchable Atrous Convolution

**Figure 4:**
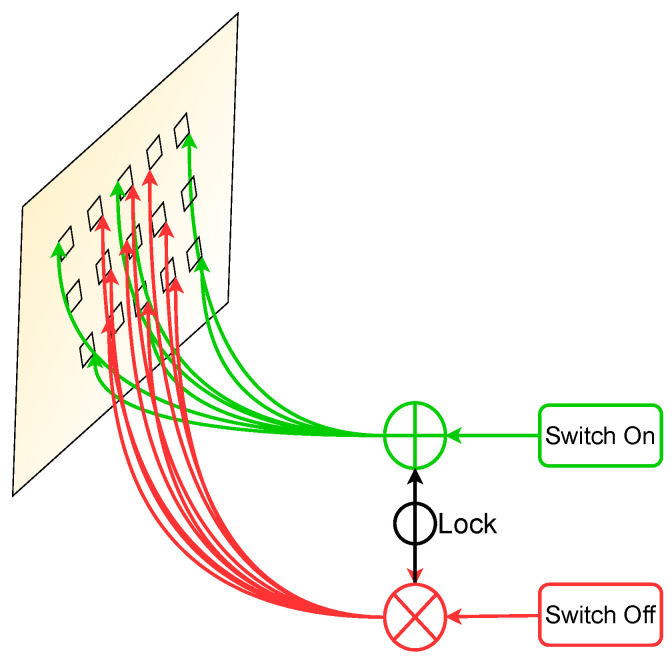
Illustrating Switchable Atrous Convolution. The red symbol ⨂ depicts atrous convolutions with an atrous rate set to 1, whereas the green symbol ⨁ denotes an atrous rate of 2 in a 3 × 3 convolutionalayer.

We replace the conventional convolutions present in backbone network ResNet [62] and FPN with SAC. The atrous convolution also referred to as dilated convolution [63] enables the ability to increase the size of effective receptive field by introducing an atrous rate. For an atrous rate of *l* in atrous convolution, it adds l−1 zeros between the values of consecutive filter. Due to this, the kernel with a size of k×k filter enlarges to a size of k+(k−1)(l−1) without causing any change in the number of network parameters. Figure 4 depicts an example of a 3×3 atrous convolution with the atrous rate of 1 (displayed in red), whereas an atrous rate of 2 is demonstrated in green color.

To transform a convolutionalayer to SAC, we employ the basic atrous convolutional operation Con that takes input *i*, weights *w*, and an atrous rate *l* and outputs *y*. Mathematically, it is given by:

(4) y=Con(i,w,).

In case of SAC explained in Reference [24], the above convolutionalayer converts into:

(5) $$Con\left(i,w,1\right)\overset{SAC}{\to }S\left(i\right)\;.\;Con\left(i,w,\right)+\left(1-S\left(i\right)\right)\;.\;Con\left(i,w+\Delta w,\right),$$

where S(.) defines the switch function which is implemented is a combination of an average pooling and convolutionayer with kernel of 5×5 and 1×1, respectively. The symbol Δw is trainable weight, and *l* is a hyper-parameter. Owing to switch function, our backbone network adapts to arbitrary scales of tabular images, defying the need for deformable convolutions [53]. We empirically set the atrous rate, *l* to 3 in our experiments. Moreover, we implement the idea ofocking mechanism [24] by setting the weights to w+Δw in order to exploit the backbone network pre-train on MS-COCO dataset [64]. Initially, Δw=0, and *w* is set according to the pre-trained weights. We refer readers to Reference [24] for a detailed explanation on SAC.

### 3.3. Cascade Mask R-CNN

To investigate the effectiveness of Recursive Feature Pyramid (RFP) and Switchable Atrous Convolution (SAC) modules on the task of table detection in scanned document images, we fuse these components into a cascade Mask R-CNN. The cascade Mask R-CNN is a direct combination of Mask R-CNN [55] and a recently proposed Cascade R-CNN [25].

**Figure 5:**
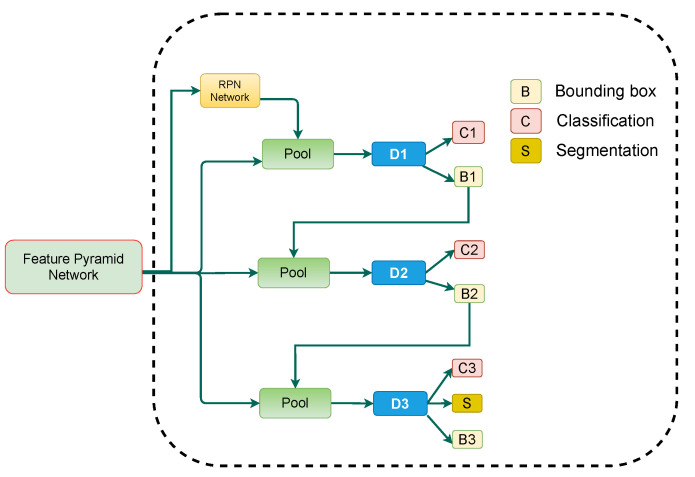
Explained architecture of Cascade Mask R-CNN module employed in the proposed pipeline. The dotted boundary outlines the two-stage detection phase of Cascade Mask R-CNN.

As depicted in Figure 5, the architecture of our utilized cascade Mask R-CNN closely follows the cascaded architecture introduced in Reference [25], along with the addition of segmentation branch at the final network head [55]. The proposed CasTabDetectoRS consists of three detectors operating on rising IoU (Intersection over Union) thresholds of 0.5, 0.6, and 0.7, respectively. The Region of Interest (ROI) pooling takesearned proposals from the Region proposal Network (RPN) and propagates the extracted ROI features to a series of network heads. The first network head receives the ROI features and performs classification and regression. The output of the first detector is treated as an input for the subsequent detector. Therefore, the predictions from the deeper network are refined andess prone to produce false positives. Furthermore, each regressor is enhanced with theocalization distribution estimated by the previous regressor instead of the actual initial distribution. This enables the network head operating on a higher IoU threshold to predict optimallyocalized bounding boxes. In the final stage of cascaded networks, along with regression and classification, the network performs segmentation to advance the final predictions further.

As illustrated in Figure 2, the proposed CasTabDetectoRS employs ResNet-50 [62] as a backbone network. Theightweight ResNet-50 backbone equipped with SAC generates feature maps from the input scanned document image. The extracted feature maps are passed to the RFP that optimally transforms the features byeveraging feedback connections. Subsequently, these optimized features are passed to the RPN that estimates the potential candidate regions of interest. In the first stage of cascade R-CNN, the network head takes the proposals from RPN and feature maps from the FPN module and performs regression and classification with an IoU threshold of 0.5. The subsequent stages of Cascade Mask R-CNN further refine the predicted bounding boxes with an increasing IoU threshold. Analogous to Reference [55], the network in the final cascaded stage segments the object in a bounding box, along with classification and regression.

## 4. Experimental Results

### 4.1. Datasets

#### 4.1.1. ICDAR-17 POD

**Figure 6:**
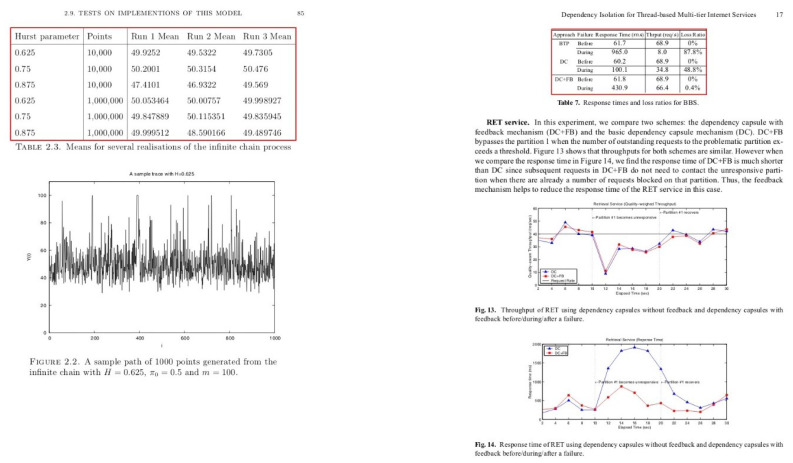
Sample document images from the ICDAR-17 POD dataset [1]. The red boundary represents the tabular area in document images.

The competition about detecting graphical Page Object Detection (POD) [1] was organized at ICDAR in 2017, which yielded the ICDAR-2017 POD dataset. The dataset contains bounding box information for tables, formulas, and figures. From 2417 images present in the dataset, 1600 images are used to fine-tune our network, and 817 images are utilized as a test set. Since the previous methods [12,20,30] have reported results on varying IoU thresholds, we present our results with an IoU threshold value ranging from 0.5–0.9 to draw a direct comparison with prior methods. A couple of samples from this dataset are illustrated in Figure 6.

#### 4.1.2. ICDAR-19

**Figure 7:**
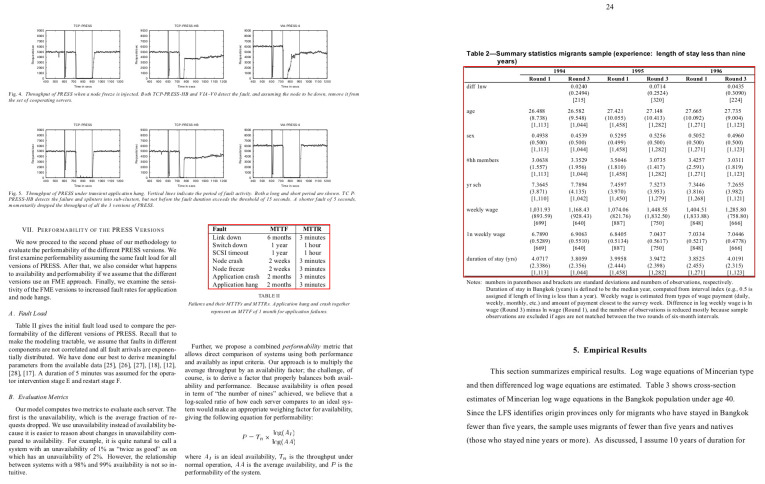
Sample document images from the ICDAR 19 Track A (Modern) dataset [65]. The red boundary highlights the tabular area in document images.

Another competition for Table Detection and Recognition (cTDaR) [65] is organized at ICDAR in 2019. For the task of table detection (TRACK A), two new datasets (historical and modern) are introduced in the competition. The historical dataset comprises hand-written accountingedgers, train timetables, whereas the modern dataset consists of scientific papers, forms, and commercial documents. In order to have a direct comparison against prior state-of-the-art [11], we report results on the modern datasets with an IoU threshold ranging from 0.5–0.9. Figure 7 depicts a pair of instances from this dataset.

#### 4.1.3. TableBank

**Figure 8:**
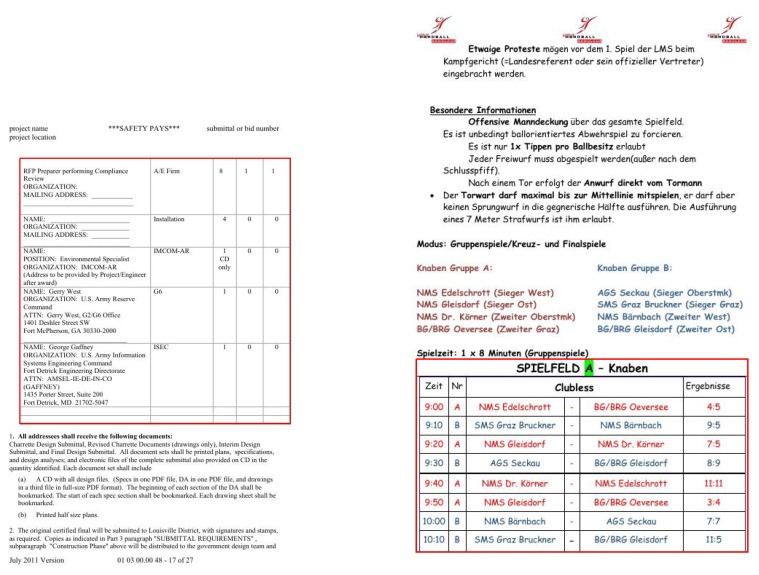
Sample document images from the TableBank dataset [66]. The red boundary outlines the tabular area in document images.

Currently, TableBank [66] is one of the enormous datasets publicly available for the task of table detection in document images. The dataset comprises 417K annotated document images that are obtained by crawling documents from the arXiv database. It is important to highlight that we take 1500 images from the splits of Word and LaTeX and 3000 samples from Word + LaTeX split. This enables our results to have a straightforward comparison with earlier state-of-the-art results [11]. For a visual aid, a couple of samples from this dataset are highlighted in Figure 8.

#### 4.1.4. UNLV

**Figure 9:**
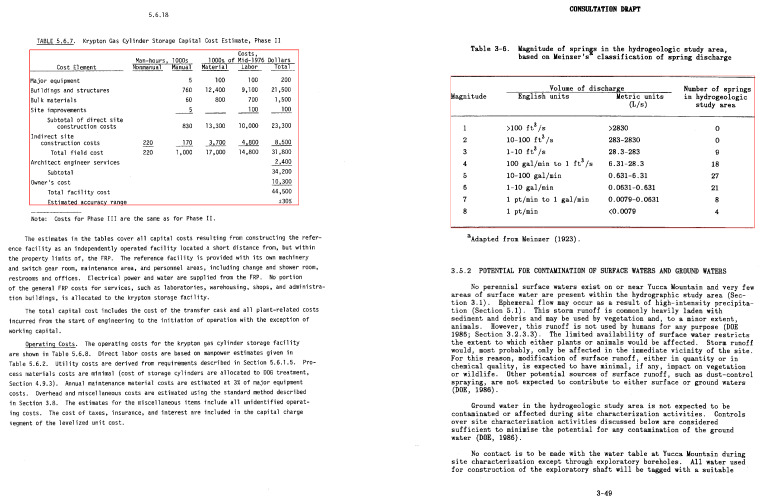
Sample document images from the UNLV dataset [67]. The red boundary marks the tabular area in document images.

UNLV [67] dataset comprises scanned document images collected from commercial documents, research papers, and magazines. The dataset has around 10K images. However, only 427 images contain tables. Since prior state-of-the-art methods [20] have only used tabular images, we follow the identical split for direct comparison. Figure 9 depicts a pair of document images from the UNLV dataset.

#### 4.1.5. Marmot

**Figure 10:**
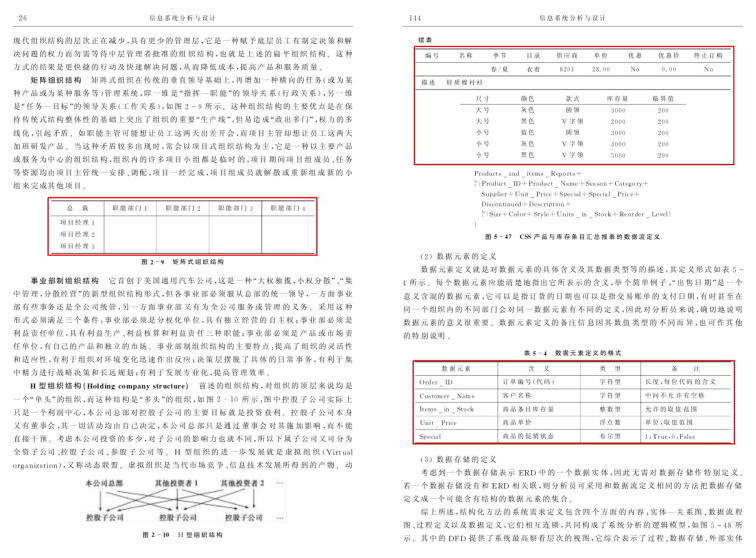
Sample document images from the Marmot dataset [68]. The red boundary denotes the tabular area in document images.

Earlier, Marmot [68] was one of the most widely exploited datasets in the table community. This dataset is published by the Institute of Computer Science and Technology (Peking University) by collecting samples from Chinese and English conference papers. The dataset consists of 2K images with an almost 1:1 ratio between positive to negative samples. For direct comparison with previous work [20], we used the cleaned version of the dataset by Reference [7] and did not incorporate any sample of the dataset in the training set. A couple of instances from the Marmot dataset are outlined in Figure 10.

### 4.2. Implementation Details

We implement CasTabDetectoRS in Pytorch byeveraging the MMdetection framework [69]. Our table detection method operates on ResNet-50 backbone network [62] pre-trained on ImageNet [70]. Furthermore, we transform all the 3×3 conventional convolutions present in the bottom-up backbone network to SAC. We closely follow the experimental configurations of Cascade Mask R-CNN [25] in order to execute the training process. All input documents images are resized with a maximum size of 1200 × 800 by preserving the actual aspect ratio. We train all the models for straight 14 epochs by initially setting theearning rate of 0.0025 with aearning rate decay of 0.1 after six epochs and ten epochs. We set the IoU threshold values to [0.5, 0.6, 0.7], respectively, for the three stages of R-CNN. We use a single anchor scale of 8, whereas the anchor ratios are set to [0.5, 1.0, 2.0]. We train all the models with a batch size of 1. We train all the models on NVIDIA GeForce RTX 1080 Ti GPU with 12 GB memory (Santa Clara, CA, USA).

### 4.3. Evaluation Protocol

Analogous to the prior table detection method on scanned documentimages [7,8,11,12,19,20,30,31,32,33], we assess the performance of our CasTabDetectoRS on precision, recall, and F1-score. We have reported the IoU threshold values, along with the achieved results for direct comparison with the existing approaches.

#### 4.3.1. Precision

The precision [71] computes the ratio of true positive samples over the total predicted samples. Mathematically, it is calculated as:

(6) $$\mathrm{Precision}=\frac{\mathrm{True}\;\mathrm{Positives}}{\mathrm{True}\;\mathrm{Positives}\;+\;\mathrm{False}\;\mathrm{Positives}.}$$

#### 4.3.2. Recall

The recall [71] is defined as the ratio of true positives over all all correct samples from the ground truth. It is calculated as:

(7) $$\mathrm{Recall}=\frac{\mathrm{True}\;\mathrm{Positives}}{\mathrm{True}\;\mathrm{Positives}\;+\;\mathrm{False}\;\mathrm{Negatives}.}$$

#### 4.3.3. F1-Score

The F1-score [71] is defined as the harmonic mean of precision and recall. Mathematically, it is given by:

(8) $$F1-\mathrm{score}=\frac{2\times \;\mathrm{Precision}\;\times \;\mathrm{Recall}}{\mathrm{Precision}\;+\;\mathrm{Recall}.}$$

#### 4.3.4. Intersection over Union

Intersection over Union (IoU) [72] computes the intersecting region between the predicted and the ground truth region. The formula for the calculation of IoU is:

(9) $$\mathrm{IoU}(A,B)=\frac{\mathrm{Area}\;\mathrm{of}\;\mathrm{Overlap}\;\mathrm{region}}{\mathrm{Area}\;\mathrm{of}\;\mathrm{Union}\;\mathrm{region}}=\frac{\left|A\cap B\right|}{\left|A\cup B\right|}.$$

### 4.4. Result and Discussion

To evaluate the performance of the proposed CasTabDetectoRS, we report the results on five different publicly available table detection datasets. This section presents a comprehensive quantitative and qualitative analysis of our presented approach on all the datasets.

#### 4.4.1. ICDAR-17 POD

**Figure 11:**
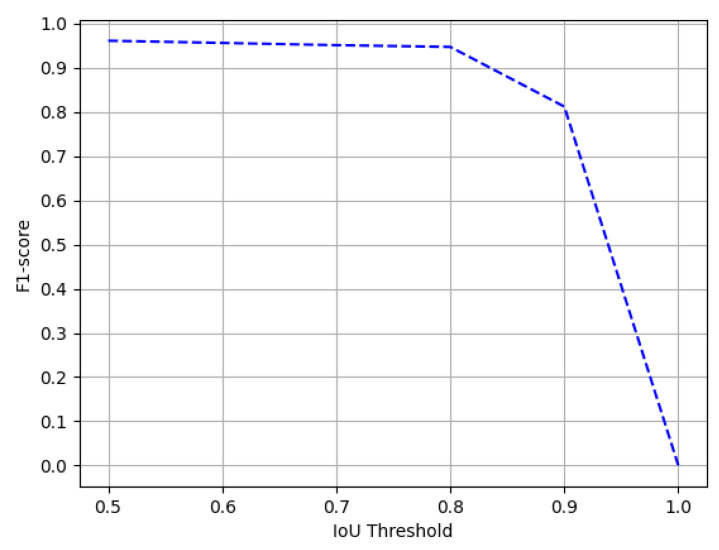
Performance evaluation of our CasTabDetectoRS in terms of F1-score over the varying IoU thresholds ranging from 0.5 to 1.0 on the ICDAR-2017-POD table detection dataset.

**Figure 12:**
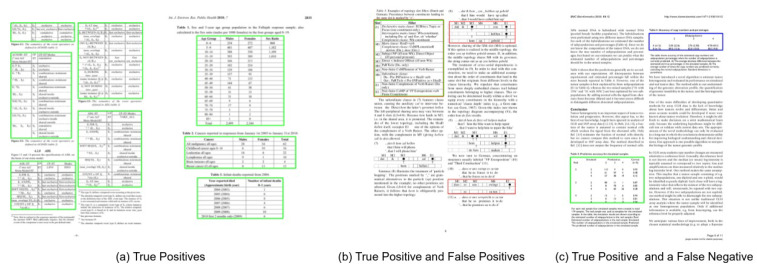
CasTabDetectoRS results on the ICDAR-2017 POD table detection dataset. Green represents true positive, red denotes false positive, and blue color highlights false negative. In this figure, (**a**) represents a couple of samples containing true positives, (**b**) highlights true positive and false positives, and (**c**) depicts a true positive and a false negative.

The ICDAR-17 POD challenge dataset consists of 817 images with 317 tables in the test set. For direct comparison with previous entries in the competition [1] and previous state-of-the-art results, we report the results on the IoU threshold value of 0.6 and 0.8. Table 1 summarizes the results achieved by our model. On an IoU threshold value of 0.6, our CasTabDetectoRS achieves a precision of 0.941, recall of 0.972, and F1-score of 0.956. On increasing the IoU threshold from 0.6 to 0.8, the performance of our network only indicates a slight drop with a precision of 0.962, recall of 0.932, and F1-score of 0.947. Furthermore, Figure 11 illustrates the effect of various IoU thresholds on our table detection system. The qualitative performance of our proposed method on the ICDAR-17 POD dataset is highlighted in Figure 12. Analysis of incorrect results discloses that the network fails toocalize precise tabular areas or produce false positives.

##### Comparison with State-of-the-Art Approaches

Byooking at Table 1, it is evident that our network achieves comparable results with the existing state-of-the-art approaches on the ICDAR-17 POD dataset. It is important to emphasize that methods introduced in References [1,20] either rely on the heavy backbone with memory-intensive deformable convolutions [53] or are dependent on multiple pre- and post-processing methods to achieve the results. On the contrary, our CasTabDetectoRS operates on aighter weight ResNet-50 backbone with switchable atrous convolutions.

Furthermore, it is vital to mention that the system [54] that produced state-of-the-art results on this datasetearns to classify tables, figures, and equations. Byeveraging the information about other graphical page objects, such as figures and equations, their system reduces the misclassification of tables. On the contrary, the proposed system only trains on theimited tabular information and has no idea about other similar graphical page objects. Therefore, havingow inter-class variance between the different graphical page objects and tables in this dataset, our network produces more false positives and fails to surpass state-of-the-art results on this dataset.

#### 4.4.2. ICDAR-19

**Figure 13:**
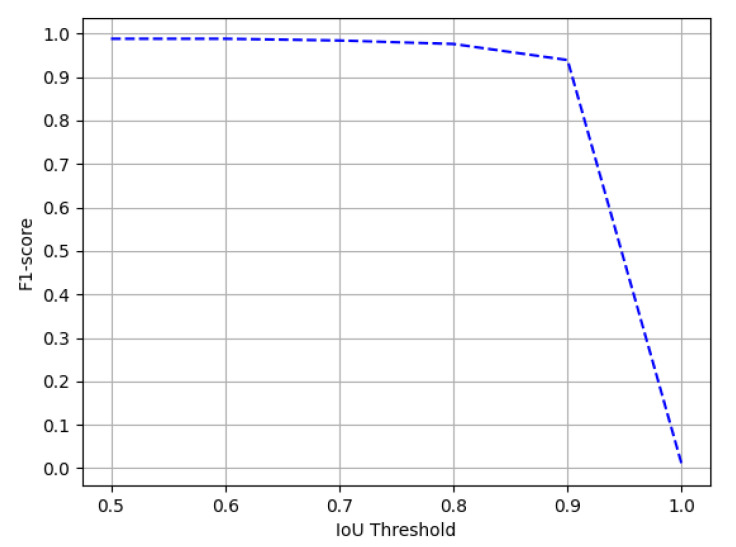
Performance evaluation of our CasTabDetectoRS in terms of F1-score over the varying IoU thresholds ranging from 0.5 to 1.0 on the ICDAR-2019 Track A (Modern) dataset.

**Figure 14:**
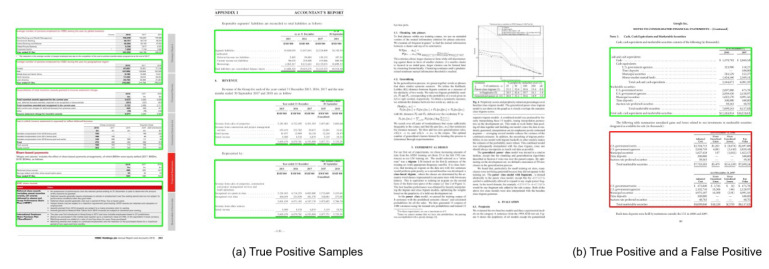
CasTabDetectoRS results on the table detection dataset of ICDAR-2019 Track A (Modern). Green represents true positive, whereas red denotes false positive. In this figure, (**a**) highlights a couple of samples containing true positives, whereas (**b**) represents a true positive and a false positive.

In this paper, the ICDAR-19 represents the Modern Track A part of the table detection dataset introduced in the table detection competition at ICDAR 2019 [65]. In order to draw strict comparisons with participants of the competition and existing state-of-the-art results, we evaluate the performance of our proposed method on the higher IoU threshold of 0.8 and 0.9. Table 2 presents the quantitative analysis of our proposed method, whereas the performance in terms of F1-score of our table detection method on various IoU thresholds is illustrated in Figure 13. The qualitative analysis is demonstrated in Figure 14. After analyzing false positives yielded by our network, we realize that the ground truth of the ICDAR-19 dataset has unlabeled tables present in the modern document images. One instance of such a scenario is exhibited in Figure 14b.

##### Comparison with State-of-the-Art Approaches

Along with presenting our achieved results on the ICDAR-19 dataset, Table 2 compares the performance of our CasTabDetectoRS with the prior state-of-the-art approaches. It is evident that our introduced cascade network equipped with RFP and SAC surpassed the previous state-of-the-art results with a significant margin. We accomplish a precision of 0.964, recall of 0.988, and an F1-score of 0.976 on an IoU threshold of 0.8. Upon increasing the IoU threshold to 0.9, the proposed table detection method achieves a precision of 0.928, recall of 0.951, and F1-score of 0.939. The higher difference between the F1-score of our method and the previously achieved F1-score clearly exhibits the superiority of our CasTabDetectoRS.

#### 4.4.3. TableBank

**Figure 15:**
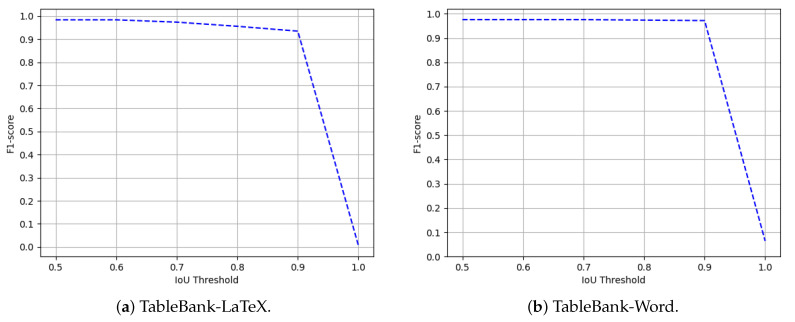
Performance evaluation of our CasTabDetectoRS in terms of F1-score over the varying IoU thresholds ranging from 0.5 to 1.0 on the TableBank-LaTeX and TableBank-Word datasets.

**Figure 16:**
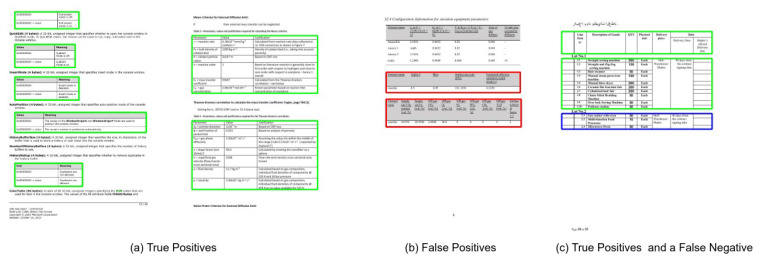
CasTabDetectoRS results on the TableBank dataset. Green represents true positive, red denotes false positive, and blue color highlights false negative. In this figure, (**a**) represents a couple of samples containing true positives, (**b**) illustrates false positives, and (**c**) depicts true positives and false negatives.

**Figure 17:**
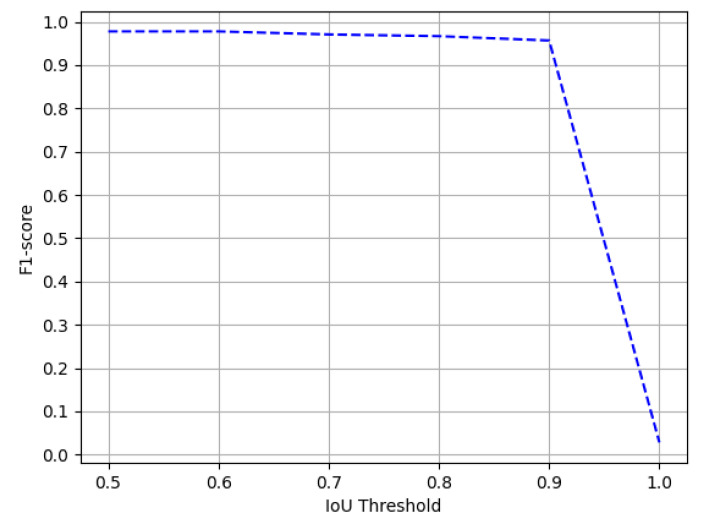
Performance evaluation of our CasTabDetectoRS in terms of F1-score over the varying IoU thresholds ranging from 0.5 to 1.0 on the TableBank-Both dataset.

We evaluate the performance of the proposed method on all the three splits of TableBank dataset [66]. To establish a straightforward comparison with the recently achieved state-of-the-art results [11] on TableBank, we report the results on the IoU threshold of 0.5. Furthermore, owing to the superior predictions of our proposed method, we present results on a higher IoU threshold of 0.9. Table 3 summarizes the performance of our CasTabDetectoRS on the splits of TableBank-LaTeX, TableBank-Word, and TableBank-Both. Along with the quantitative results, we demonstrate the performance of the proposed system in terms of F1-score by increasing the IoU thresholds from 0.5 to 1.0. Figure 15 depicts the drop in performance on the split of TableBank-LaTeX and TableBank-Word, whereas, Figure 16 depicts a couple of true positives and one instance each of false positive and a false negative. Figure 17 explains the F1-score on the split of TableBank-Both dataset.

##### Comparison with State-of-the-Art Approaches

Table 3 provides the comparison between existing state-of-the-art table detection methods and our proposed approach. It is clear that our proposed CasTabDetectoRS has surpassed the previous baseline and state-of-the-art methods on all the three splits of the TableBank dataset. On the dataset split of TableBank-LaTeX, we achieve an F1-score of 0.984 and 0.935 with an IoU threshold of 0.5 and 0.9, respectively. Similarly, we accomplish F1-scores of 0.976 and 0.972 on the IoU threshold of 0.5 and 0.9, respectively, on the TableBank-Word dataset. Moreover, we attain F1-scores of 0.978 and 0.957 on IoU of 0.5 and 0.9, respectively, on the TableBank-(Word + LaTex) dataset.

#### 4.4.4. Marmot

**Figure 18:**
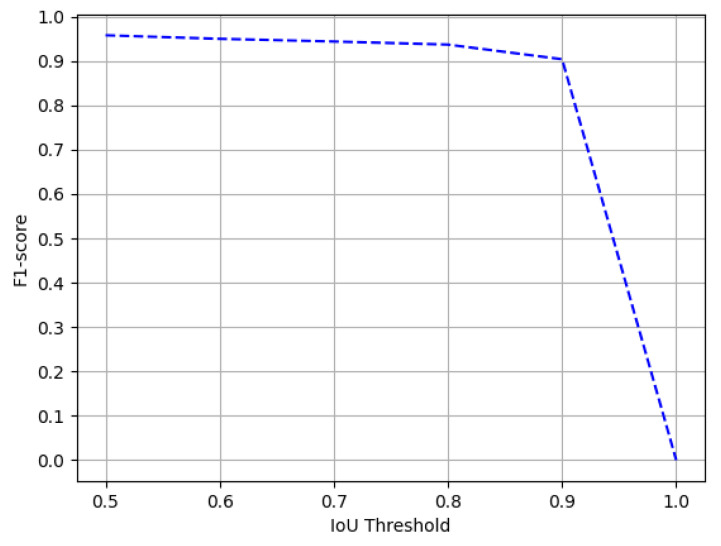
Performance evaluation of our CasTabDetectoRS in terms of F1-score over the varying IoU thresholds ranging from 0.5 to 1.0 on the Marmot dataset.

**Figure 19:**
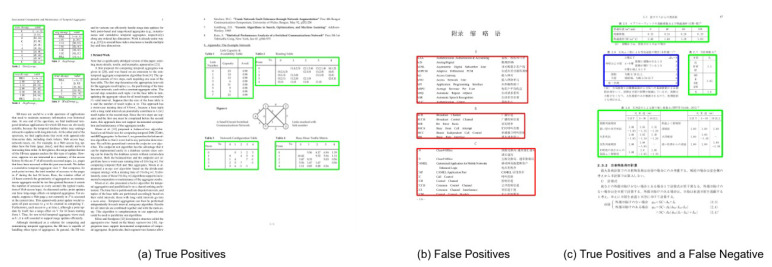
CasTabDetectoRS results on the Marmot dataset. Green represents true positive, red denotes false positive, and blue color highlights false negative. In this figure, (**a**) exhibits a couple of samples containing true positives, (**b**) illustrates false positives, and (**c**) depicts true positives and false negatives.

The Marmot dataset consists of 1967 document images comprising 1348 tables. Since prior state-of-the-art approaches [12,20] have employed the model trained on the ICDAR-17 dataset to evaluate the performance on the Marmot dataset, we have identically reported the results to have a direct comparison. Table 4 presents the quantitative analysis of our proposed method, whereas Figure 18 illustrates the effect of our CasTabDetectoRS on increasing the IoU threshold from 0.5 to 1.0. Figure 19 portrays the qualitative assessment of our table detection system on the Marmot dataset by illustrating samples of true positives, false positives, and a false negative.

##### Comparison with State-of-the-Art Approaches

Table 4 summarizes the performance comparison between the previous state-of-the-art results and the results achieved by our CasTabDetectoRS Marmot dataset. Our proposed method outperforms the previous results with an F1-score of 0.958 and 0904 on the IoU threshold values of 0.5 and 0.9, respectively.

#### 4.4.5. UNLV

**Figure 20:**
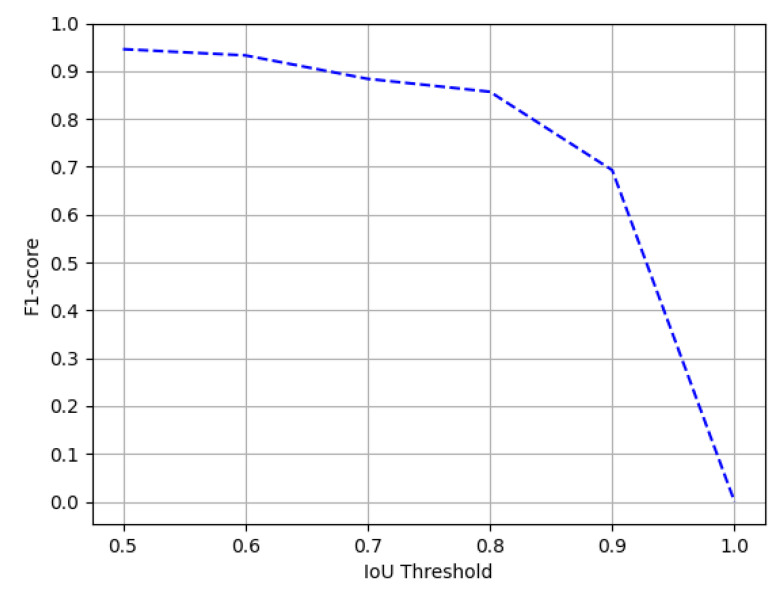
Performance evaluation of our CasTabDetectoRS in terms of F1-score over the varying IoU thresholds ranging from 0.5 to 1.0 on the UNLV dataset.

**Figure 21:**
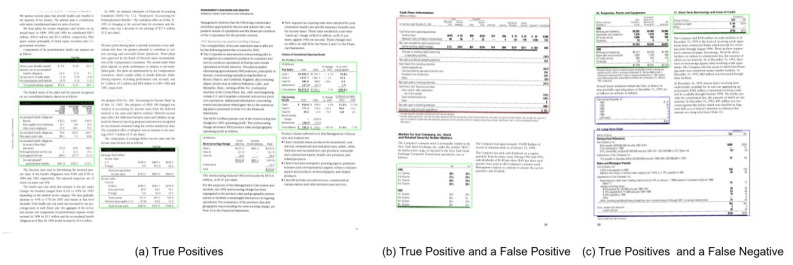
CasTabDetectoRS results on the UNLV dataset. Green represents true positive, red denotes false positive, and blue color highlights false negative. In this figure, (**a**) highlights a couple of samples containing true positives, and (**b**) represents a true positive and a false positive, whereas (**c**) depicts true positives and false negatives.

The UNLV dataset comprises 424 document images containing a total of 558 tables. We evaluate the performance of our presented method on the UNLV dataset to exhibit the completeness of our approach. Similarly, for direct comparison with prior works [12,19] on this dataset, we present our results on the IoU threshold of 0.5 and 0.6 as summarized in Table 5. Moreover, Figure 20 explains the deterioration in performance of the system on increasing the IoU threshold from 0.5 to 1.0. For the qualitative analysis on the UNLV dataset, examples of true positives, false positives, and a false negative are illustrated in Figure 21.

##### Comparison with State-of-the-Art Approaches

The performance comparison between the proposed method and previous attempts on the UNLV dataset is summarized in Table 5. With the obtained results, it is apparent that our proposed system has outsmarted earlier methods with F1-scores of 0.946 and 0.933 on the IoU threshold values of 0.5 and 0.6, respectively.

#### 4.4.6. Cross-Datasets Evaluation

Currently, the deepearning-based table detection methods are preferred over rule-based methods due to their better generalization capabilities over distinctive datasets. To investigate how well our proposed CasTabDetectoRS generalize over different datasets, we perform cross-dataset evaluation by incorporating four state-of-the-art table detection models inferred over five different datasets. We summarize all the results in Table 6.

With the table detection model trained on the TableBank-LaTeX dataset, apart from ICDAR-19, we achieve impressive results on ICDAR-17, TableBank-Word, Marmot, and UNLV with an average F1-score of 0.865. After manual inspection, we observe that the system produces several false positives due to the varying nature of document images in ICDAR-19 and TableBank-LaTeX. The table detection model trained on the ICDAR-17 dataset yields the average F1-score of 0.812 owing to the poor results achieved on the ICDAR-19 and UNLV datasets. The network trained on the ICDAR-19 dataset becomes the most generalized model accomplishing the average F1-score of 0.924. Although the size of the UNLV dataset is small (424 document images), the model trained on this dataset generates second-best results with an average F1-score of 0.897.

Manual investigation of cross-datasets evaluation yields the misinterpretation of other graphical page objects [2] with tables. However, with the obtained results, it is evident that our proposed CasTabDetectoRS produces state-of-the-art results on a specific dataset and generalizes well over the other datasets. Such types of well-generalized table detection systems for scanned document images are required in several domains [8].

## 5. Conclusions and Future Work

This paper presents CasTabDetectoRS, the novel table detection framework for scanned document images, which comprises Cascade Mask R-CNN with a Recursive Feature Pyramid (RFP) network with Switchable Atrous Convolutions (SAC). The proposed CasTabDetectoRS accomplishes state-of-the-art performances on the four different table detection datasets (ICDAR-19 [65], TableBank [66], UNLV [67], and Marmot [68]), while achieving comparable results on the ICDAR-17-POD [1] dataset.

Upon direct comparison against previous state-of-the-art results on ICDAR-19 Track A (Modern) dataset, we reduce the relative error by 56.36% and 29.89% in terms of achieved F1-score on IoU thresholds of 0.8 and 0.9, respectively. On the dataset of TableBank-LaTeX and TableBank-Word, we decrease the relative error by 20% on each dataset split. On TableBank-Both, we reduce the relative error by 12%. Similarly, on the Marmot dataset [68], we observe a 4.55% reduction, whereas the system achieves a relative error reduction of 3.5% on the UNLV dataset [67]. Furthermore, this paper empirically establishes that, instead of incorporating heavy backbone networks [11,12] and memory exhaustive deformable convolutions [20], state-of-the-art results are achievable by employing a relativelyightweight backbone network (ResNet-50) with SAC. Moreover, this paper demonstrates the generalization capabilities of the proposed CasTabDetectoRS through extensive cross-datasets evaluations. It is important to emphasize that our proposed network takes 9.9 gigabytes of VRAM (Video Read Access Memory) memory with an inference time of 10.8 frames per second. The achieved network complexity is incomparable since prior state-of-the-art methods in this domain have not reported their network complexity and inference time.

In the future work, we plan to extend the proposed framework by tackling the even more challenging task of table structure recognition in scanned document images. We expect that our cross-datasets evaluation sets a benchmark that will be followed in future examinations of table detection methods. Furthermore, the backbone network and the region proposal network of the proposed pipeline can be enhanced by exploiting the attention mechanism [73,74].

## Figures and Tables

**Table 1:** Performance comparison between the proposed CasTabDetectoRS and previous state-of-the-art results on table detection dataset of ICDAR-17 POD. Best results are highlighted in the table.

Method	IoU = 0.6			IoU = 0.8		
	Recall	Precision	F1-Score	Recall	Precision	F1-Score
DeCNT [20]	0.971	0.965	0.968	0.952	0.946	0.949
NLPR-PAL [1]	0.953	0.968	0.960	**0.958**	0.943	0.951
VisInt [1]	0.918	0.924	0.921	0.823	0.829	0.826
GOD [54]	-	-	**0.989**	-	-	**0.971**
CDeC-Net [12]	0.931	**0.977**	0.954	0.924	**0.970**	0.947
HybridTabNet [14]	**0.997**	0.882	0.936	**0.994**	0.879	0.933
CasTabDetectoRS (Ours)	0.941	0.972	0.956	0.932	0.962	0.947

**Table 2:** Performance comparison between the proposed CasTabDetectoRS and previous state-of-the-art results on the dataset of ICDAR 19 Track A (Modern). Best results are highlighted in the table.

Method	IoU = 0.8			IoU = 0.9		
	Recall	Precision	F1-Score	Recall	Precision	F1-Score
TableRadar [65]	0.940	0.950	0.945	0.890	0.900	0.895
NLPR-PAL [65]	0.930	0.930	0.930	0.860	0.860	0.860
Lenovo Ocean [65]	0.860	0.880	0.870	0.810	0.820	0.815
CascadeTabNet [11]	-	-	0.925	-	-	0.901
CDeC-Net [12]	0.934	0.953	0.944	0.904	0.922	0.913
HybridTabNet [14]	0.933	0.920	0.928	0.905	0.895	0.902
**CasTabDetectoRS (Ours)**	**0.988**	**0.964**	**0.976**	**0.951**	**0.928**	**0.939**

**Table 3:** Performance comparison between the proposed CasTabDetectoRS and previous state-of-the-art results on various splits of TableBank dataset. The double horizontalines divide the different splits. Best results are highlighted in the table.

Method	Dataset	IoU = 0.5			IoU = 0.9		
		Recall	Precision	F1-Score	Recall	Precision	F1-Score
CascadeTabNet [11]	TableBank-LaTeX	0.972	0.959	0.966	-	-	-
Li et al. [66]	TableBank-LaTeX	0.962	0.872	0.915	-	-	-
HybridTabNet [14]	TableBank-LaTeX	-	-	0.980	-	-	0.934
**CasTabDetectoRS (Ours)**	TableBank-LaTeX	**0.984**	**0.983**	**0.984**	**0.935**	**0.935**	**0.935**
CascadeTabNet [11]	TableBank-Word	0.955	0.943	0.949	-	-	-
Li et al. [66]	TableBank-Word	0.803	0.965	0.877	-	-	-
HybridTabNet [14]	TableBank-Word	-	-	0.970	-	-	0.962
**CasTabDetectoRS (Ours)**	TableBank-Word	**0.985**	**0.967**	**0.976**	**0.981**	**0.963**	**0.972**
CascadeTabNet [11]	TableBank-Both	0.957	0.944	0.943	-	-	-
Li et al. [66]	TableBank-Both	0.904	0.959	0.931	-	-	-
HybridTabNet [14]	TableBank-Both	-	-	0.975	-	-	0.949
**CasTabDetectoRS (Ours)**	TableBank-Both	**0.982**	**0.974**	**0.978**	**0.961**	**0.953**	**0.957**

**Table 4:** Performance comparison between the proposed CasTabDetectoRS and previous state-of-the-art results on the Marmot dataset. Best results are highlighted in the table.

Method	IoU = 0.5			IoU = 0.9		
	Recall	Precision	F1-Score	Recall	Precision	F1-Score
DeCNT [20]	0.946	0.849	0.895	-	-	-
CDeC-Net [12]	0.930	0.975	0.952	0.765	0.774	0.769
HybridTabNet [14]	0.961	0.951	0.956	0.903	0.900	0.901
**CasTabDetectoRS (Ours)**	**0.965**	**0.952**	**0.958**	**0.901**	**0.906**	**0.904**

**Table 5:** Performance comparison between the proposed CasTabDetectoRS and previous state-of-the-art results on the UNLV dataset. Best results are highlighted in the table.

Method	IoU = 0.5			IoU = 0.6		
	Recall	Precision	F1-Score	Recall	Precision	F1-Score
Gilani et al. [19]	0.907	0.823	0.863	-	-	-
CDeC-Net [12]	0.906	0.914	0.910	0.805	0.961	0.883
HybridTabNet [14]	0.926	0.962	0.944	**0.914**	0.949	0.932
**CasTabDetectoRS (Ours)**	**0.928**	**0.964**	**0.946**	**0.914**	**0.952**	**0.933**

**Table 6:** Examining the generalization capabilities of the proposed CasTabDetectoRS through cross datasets evaluation.

Training Dataset	Testing Dataset	Recall	Precision	F1-Score	Average F1-Score
TableBank-LaTeX	ICDAR-19	0.605	0.778	0.680	0.865
	ICDAR-17	0.866	0.958	0.910	
	TableBank-Word	0.967	0.947	0.957	
	Marmot	0.893	0.963	0.927	
	UNLV	0.918	0.856	0.885	
ICDAR-17	ICDAR-19	0.649	0.778	0.686	0.812
	TableBank-Word	0.983	0.943	0.963	
	Marmot	0.965	0.952	0.958	
	UNLV	0.607	0.685	0.644	
ICDAR-19	ICDAR-17	0.894	0.917	0.906	0.924
	TableBank-Word	0.981	0.921	0.950	
	Marmot	0.925	0.956	0.940	
	UNLV	0.898	0.876	0.887	
UNLV	ICDAR-17	0.867	0.879	0.881	0.897
	TableBank-Word	0.903	0.941	0.922	
	Marmot	0.874	0.945	0.908	
	ICDAR-19	0.839	0.918	0.877	

## Data Availability

Not applicable.
